# Changes in diaphragmatic excursion and lung compliance during gynaecologic surgery: open laparotomy versus laparoscopy—a prospective observational study

**DOI:** 10.1038/s41598-020-78375-2

**Published:** 2020-12-08

**Authors:** Kyungmi Kim, Kyoung-Sun Kim, A. Rom Jeon, Jong-Yeon Park, Woo-Jong Choi

**Affiliations:** Laboratory for Cardiovascular Dynamics, Department of Anesthesiology and Pain Medicine, Asan Medical Center, University of Ulsan College of Medicine, 88, Olympic-ro 43-gil, Songpa-gu, Seoul, 05505 Republic of Korea

**Keywords:** Medical research, Risk factors

## Abstract

This study compared the effects of open versus laparoscopic radical hysterectomy on intraoperative diaphragmatic excursion and lung compliance. We enrolled 20 women per group; Group O’s members underwent open radical hysterectomy, while Group L’s members underwent laparoscopic radical hysterectomy. Diaphragmatic excursion was measured by assessing tidal ventilation using M-mode ultrasonography before intubation (T0), after intubation with mechanical ventilation (T1), 90 min after incision (T2), and at the end of the operation with recovery of muscle relaxation (T3). Peak inspiratory pressure and static lung compliance were measured using an anaesthesia machine combined with a ventilator. Diaphragmatic excursion was significantly lower in Group L than in Group O at T2 (5.3 ± 1.7 mm vs. 7.7 ± 2.0 mm, P < 0.001) and T3 (8.4 ± 1.9 vs. 10.4 ± 2.4, P = 0.011). Impaired diaphragmatic excursion at T3 (< 10 mm under mechanical ventilation) occurred in 15 patients (83.3%) in Group L and seven (38.9%) in Group O (P = 0.006). Changes over time in peak inspiratory pressure and static lung compliance differed significantly between the two groups (P < 0.001 each). Laparoscopic radical hysterectomy decreased diaphragmatic excursion and static lung compliance significantly more than open radical hysterectomy.

**Korean clinical trial number:** Korean Clinical Trials Registry (KCT0004477) (Date of registration: November 18 2019) (https://cris.nih.go.kr/cris/search/search_result_st01_en.jsp?seq=14963&ltype=&rtype=).

## Introduction

Laparoscopic surgery is generally preferred to open abdominal surgery because the former is associated with a lower incidence of pulmonary complications and a shorter hospital stay^1,2^. However, we previously reported that laparoscopic radical hysterectomy was associated with the development of impaired diaphragmatic excursion at the end of the operation^3^. Our previous results suggested that laparoscopic radical hysterectomy aggravated physiological changes in pulmonary parameters and worsened diaphragmatic excursions. Laparoscopic radical hysterectomy requires the steep Trendelenburg position and pneumoperitoneum, which results in cephalic displacement of the diaphragm and a reduction in diaphragmatic movement. Moreover, numerous reports have emphasised applying positive end-expiratory pressure (PEEP) and recruitment manoeuvres during laparoscopic surgery because of the Trendelenburg position and pneumoperitoneum^4–8^.

This implies that the use of laparoscopic surgery cannot guarantee zero incidences of postoperative pulmonary complications. We then encountered the difficult situation of having to decide what kind of surgery is the better option for patient safety when underlying diseases (e.g., morbid obesity, chronic obstructive pulmonary disease, and interstitial lung disease) are present that are associated with a high risk of postoperative pulmonary complications.

The present study therefore compared the impact of the type of gynaecological surgery, open versus laparoscopic radical hysterectomy, on diaphragmatic excursion and lung compliance.

## Results

The demographic characteristics of the participants are shown in Table 1. There were no significant differences between the patients who underwent open and laparoscopic radical hysterectomy.

**Table 1:** Demographic and perioperative characteristics of the study patients.

	Open (n = 18)	Laparoscope (n = 18)	*P*
Age (years)	45.5 ± 9.6	49.4 ± 9.0	0.212
Body mass index (kg/m^2^)	21.4 ± 2.5	22.8 ± 3.5	0.158
Hypertension	2 (11.1%)	2 (11.1%)	> 0.999
Diabetes mellitus	0 (0%)	0 (0%)	
Other systemic diseases	3 (16.7%)	3 (16.7%)	> 0.999
Hospital stay (days)	10.2 ± 7.3	10.2 ± 7.9	0.983
**Intraoperative data**			
Operative time (min)	184.3 ± 72.9	226.1 ± 68.0	0.085
Admitted crystalloid (ml)	1819.4 ± 940.1	2352.8 ± 789.2	0.074

Data are expressed as mean ± standard deviation or number (percentage).

**Figure 1:**
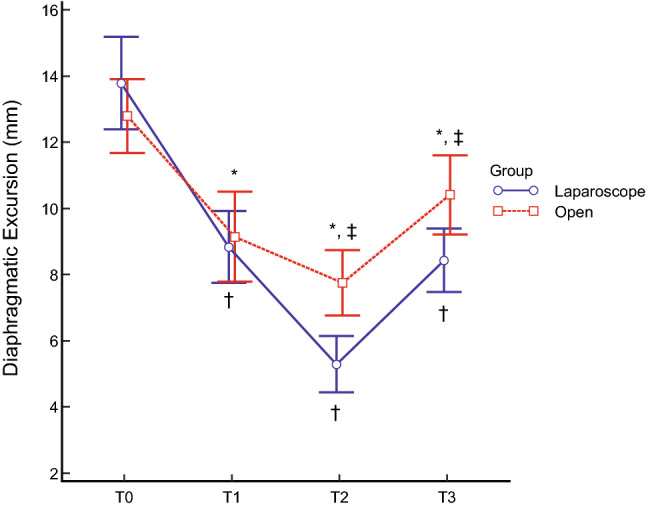
Diaphragmatic excursions at each surgical time point. Diaphragmatic movement decreased after anaesthetic induction and gradually decreased during the operation in both groups. Diaphragmatic excursions were significantly lower in patients undergoing laparoscopic (blue line) relative to those undergoing open (red line) surgery at T2 (P < 0.001) and T3 (P = 0.011). *P < 0.05 compared with T0 in patients who underwent open radical hysterectomy. ^†^P < 0.05 compared with T0 in patients who underwent laparoscopic radical hysterectomy. ^‡^Significant difference between the two groups. T0 = before intubation; T1 = after intubation; T2 = 90 min after the incision; T3 = at the end of the operation with recovery of muscle relaxation.

Despite the changes in diaphragmatic excursions not having any significant group-by-time interactions (P = 0.079), as shown in Fig. 1, the mean diaphragmatic excursions were significantly lower in patients who underwent laparoscopic radical hysterectomy at T2 (5.3 ± 1.7 mm vs 7.7 ± 2.0 mm, P < 0.001) and T3 (8.4 ± 1.9 mm vs 10.4 ± 2.4 mm, P = 0.011). Impaired diaphragmatic excursion at T3, defined as diaphragmatic excursion < 10 mm under mechanical ventilation, occurred in 15 (83.3%) patients who underwent laparoscopic radical hysterectomy and seven (38.9%) who underwent open radical hysterectomy (P = 0.006). The intra-observer correlation coefficient of measuring the diaphragmatic excursion was 0.991 (95% confidence interval 0.987–0.993, P < 0.001).

**Figure 2:**
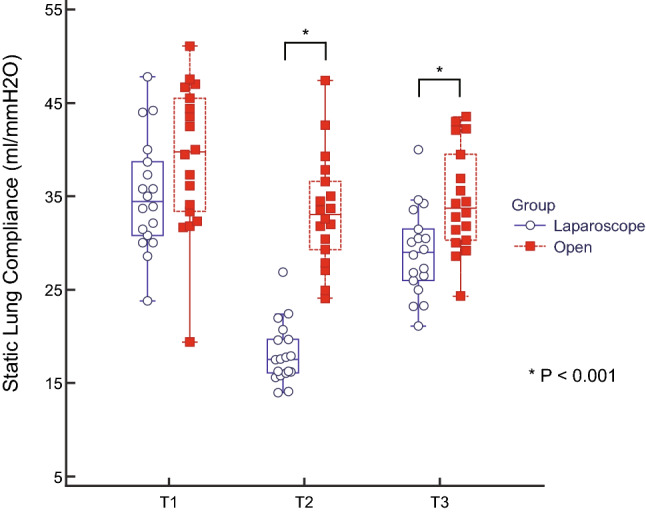
Static lung compliance during each type of surgery. Box-and-whisker plots of static lung compliance in patients who underwent laparoscopic (blue box) and open (red box) radical hysterectomy. Static lung compliance in patients who underwent laparoscopic surgery was reduced significantly during the operation and was significantly lower than in the open-surgery group at the end of the operation (P < 0.001). *The two groups differed significantly. T1 = after intubation; T2 = 90 min after the incision; T3 = at the end of the operation with recovery of muscle relaxation.

Table 2 shows the arterial blood gas analysis and pulmonary variables during the operation. Peak inspiratory pressure (PIP) was increased at T2 and slightly decreased at T3 in both groups, with the changes in PIP over time differing significantly between the two groups (P < 0.001). Plateau pressure, dynamic lung compliance and static lung compliance were significantly changed over time between the two groups (each P < 0.001). As shown in Fig. 2, the static lung compliance decreased at T2 and recovered at T3, with the changes in static lung compliance over time differing significantly between these two groups (P < 0.001).

**Table 2:** Pulmonary variables during the operation.

	Group	T1	T2	T3	*P*	
					Group × time	Intergroup
PaO_2_ on FiO_2_ 0.5 (mmHg)	Open	244.7 ± 69.3	217.2 ± 41.0	229.7 ± 26.9	0.047	0.634
	Laparoscope	240.2 ± 52.4	204.6 ± 40.2	210.6 ± 39.0		
PaCO_2_ (mmHg)	Open	36.1 ± 4.5	37.5 ± 10.4	39.1 ± 3.7	0.640	0.989
	Laparoscope	35.8 ± 4.1	40.7 ± 4.7	39.3 ± 5.5		
PIP (mmH_2_O)	Open	10.9 ± 2.4	13.1 ± 1.4	12.9 ± 1.8	< 0.001	< 0.001
	Laparoscope	12.8 ± 2.0	25.5 ± 2.8	15.9 ± 2.1		
PP (mmH_2_O)	Open	10.4 ± 2.3	12.2 ± 1.5	11.7 ± 1.5	< 0.001	< 0.001
	Laparoscope	12.4 ± 2.0	23.4 ± 2.0	15.3 ± 2.5		
Dynamic lung compliance (ml/mmH_2_O)	Open	37.4 ± 7.4	30.9 ± 4.8	31.8 ± 4.9	< 0.001	< 0.001
	Laparoscope	33.9 ± 5.0	17.2 ± 2.2	27.5 ± 3.2		
Static lung compliance (ml/mmH_2_O)	Open	39.1 ± 7.8	33.4 ± 6.0	34.6 ± 5.6	< 0.001	< 0.001
	Laparoscope	35.2 ± 6.1	18.1 ± 3.3	29.0 ± 4.7		

Data are expressed as mean ± standard deviation.

T1 = after intubation, T2 = 90 min after the incision, T3 = at the end of the operation with recovery of muscle relaxation under mechanical ventilation, FiO_2_ = fraction of inspired oxygen, PIP = peak inspiratory pressure, PP = plateau pressure. PIP, PP, dynamic and static lung compliance measured by an anaesthesia machine (Primus^Ⓡ^, Dragger, Lubeck, Germany).

Six (33.3%) patients who underwent open and seven (38.9%) who underwent laparoscopic radical hysterectomy had abnormal findings on chest X-ray within 15 postoperative days (P = 0.729). There was no patient with higher than grade II postoperative pulmonary complications.

## Discussion

The present study showed that intraoperative diaphragmatic excursion and static lung compliance were reduced more in patients who underwent laparoscopic than in those who underwent open radical hysterectomy. Moreover, impaired diaphragmatic excursion at the end of surgery occurred more frequently in patients who underwent laparoscopic than in those who underwent open radical hysterectomy.

Our previous study showed that laparoscopic radical hysterectomy reduced diaphragmatic excursion and lung compliance not only during the operation but also at the end of the surgery after neuromuscular reversal^3^. In addition, the present study found that laparoscopic radical hysterectomy worsened diaphragmatic excursion and lung compliance more than open radical hysterectomy did. Laparoscopic radical hysterectomy starts with pneumoperitoneum and requires the steep Trendelenburg position. Pneumoperitoneum leads to the cephalic displacement of the diaphragm, increments in the peak and plateau airway pressures, and a decrease in lung compliance. The steep Trendelenburg position aggravates these pulmonary mechanics during the operation. Laparoscopic radical hysterectomy, therefore, reduces diaphragmatic movement and lung compliance^7,9–11^. A comparison of intraoperative lung compliance between patients undergoing open and laparoscopic cholecystectomy showed that the reduction in compliance was significantly greater in the patients who underwent laparoscopic cholecystectomy^12^. Similarly, the present study showed that laparoscopic radical hysterectomy worsened pulmonary mechanics, with reduced lung compliance followed by decreased diaphragmatic excursion.

Open radical hysterectomy also reduces the diaphragmatic excursion and lung compliance, although to a lesser extent than laparoscopic radical hysterectomy, suggesting that general anaesthesia affects both diaphragmatic excursion and lung compliance. General anaesthesia has been reported to alter the location and movement of the diaphragm because it contains a muscle relaxant and is administered in a supine position under mechanical ventilation, reducing lung volume due to atelectasis and airway collapse^13,14^. In line with previous reports, our results showed that diaphragmatic excursion was lower at T1 than at T0 in both groups.

Despite the recovery of muscle relaxation in both groups (train-of-four [TOF] ratio > 0.9), diaphragmatic excursion at T3 did not return to the value at T0. Diaphragmatic movement has previously been shown to be significantly lower postoperatively than preoperatively^15^. Moreover, postoperative atelectasis was found to persist for 24 h after laparoscopic surgery and for 48 h after open surgery^16^. Similarly, our results showed that diaphragmatic excursion was impaired at T3, though muscle relaxation recovered, with a TOF ratio > 0.9. Although the exact mechanism of impaired diaphragmatic excursion has not been determined, the impairment might be caused by a reduction in lung volume during the operation and the anatomical peculiarity of the diaphragm, which has a C-shaped fibrous structure. Further studies are required to evaluate the mechanisms responsible for reduced diaphragmatic excursion after an operation.

Ultrasound has been reported to be reliable in assessing diaphragmatic excursion qualitatively and quantitatively^17–20^. Sonographic evaluation of the diaphragm might be a feasible method of assessing diaphragmatic function^19^. Ultrasound examinations can be performed numerous times since they expose neither the patient nor the surgeon to hazardous chemicals or radiation; they require only a few minutes to perform and are more precise in diagnosing diaphragmatic dysfunction than fluoroscopy^18,21^. Ultrasound assessment of diaphragmatic function during the intraoperative and immediate postoperative periods might become routine for evaluating patients at high risk of postoperative pulmonary complications. In addition to being useful in assessing diaphragmatic kinetics^22^, our results highlight the importance of ultrasound in evaluating the diaphragm when assessing the respiratory function.

The present study had several limitations. This observational study was performed at a single centre, with diaphragmatic excursions in all patients measured by a single examiner. The intra-observer correlation coefficient was determined after measuring diaphragmatic excursions though the inter-observer correlation coefficient cannot be confirmed. Moreover, the patients were not randomised to either laparoscopic or open radical hysterectomy. However, there were not significant differences in baseline characteristics between the two groups.

Given that laparoscopic abdominal surgery aggravates atelectasis formation and results in decreased lung compliance, the application of PEEP and recruitment manoeuvres during surgery could increase oxygenation and improve lung mechanics^4–8^. However, there was a multicentre observational study in which around 20% of patients did not receive PEEP during routine anaesthetic practice^23^. Applying PEEP may sometimes be regarded as an optional manoeuvre. Our study was designed to evaluate diaphragmatic movement only during the operation. We therefore did not assess the impact of PEEP or recruitment manoeuvres on diaphragmatic excursion, highlighting the need for further studies.

Finally, although postoperative chest X-rays were obtained within 15 days, diaphragmatic excursion on all postoperative days could not be assessed. The results of postoperative X-rays did not differ significantly between the two groups.

Even though our results cannot be generalised to all patients who undergo radical hysterectomy, they may be helpful when deciding on the type of surgery to perform for patients who have underlying lung diseases that are associated with postoperative pulmonary complications and require point-of-care during the perioperative period. Further studies are required to determine the time taken to recover from diaphragmatic impairment and to assess long-term postoperative outcomes.

In conclusions, laparoscopic radical hysterectomy decreases diaphragmatic excursion and lung compliance significantly more than open radical hysterectomy. The former procedure requires the Trendelenburg position and pneumoperitoneum. Furthermore, impaired diaphragmatic excursion at the end of surgery was more frequent in patients who underwent laparoscopic radical hysterectomy than in those who underwent open radical hysterectomy.

## Methods

### Patients

**Figure 3:**
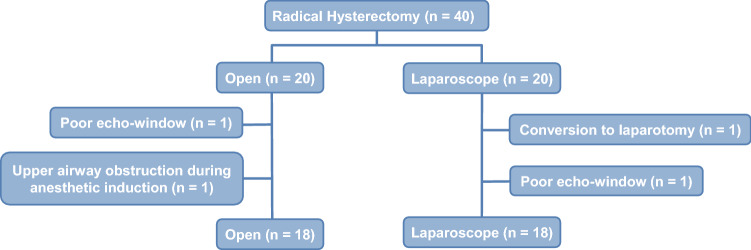
Flow diagram of patients who underwent radical hysterectomy. Forty patients were enrolled. Two patients were withdrawn from each group. Data from 36 patients were analysed.

All included patients provided written informed consent. The study protocol was approved by the Institutional Ethics Committee of the Asan Medical Center (AMC IRB 2019-0761, Seoul, Korea), and the study was registered in the Korean Clinical Trials Registry (KCT0004477). This study enrolled 20 adult patients (American Society of Anesthesiologists physical status I–II) who prospectively underwent elective open radical hysterectomy. The control group consisted of 20 patients who had previously undergone laparoscopic radical hysterectomy^3^. Patients were excluded if they had chronic obstructive pulmonary disease, respiratory dysfunction, or a body mass index > 30 kg/m^2^. Two patients who underwent open radical hysterectomy were withdrawn from this study: one with a poor echo window due to the operation field, and the other with upper airway obstruction during induction of anaesthesia. In addition, two control patients who underwent laparoscopic radical hysterectomy were withdrawn, one due to conversion to laparotomy, and the other because there was a poor echo window. Figure 3 shows that this study was performed according to the STROBE guidelines.

### Clinical data

Baseline characteristics were recorded, including patient age, body mass index, and history of systemic disease (e.g., hypertension and diabetes). Operative characteristics were recorded, including the length of the operation, the volume of administered fluids, and the results of intraoperative arterial blood gas analysis. Pulmonary mechanics, including PIP, plateau pressure, dynamic lung compliance and static lung compliance, were acquired from the anaesthesia machine (PrimusⓇ, Dragger, Lubeck, Germany) combined with a ventilator. The definition of postoperative pulmonary complications was the same as that used in a previous study by Dindo et al., which was reported in *Annals of Surgery*^24^. The electronic medical records and chest X-rays within 15 days after the surgery of study patients were using to check the postoperative pulmonary complications.

### Anaesthesia and mechanical ventilation

All anaesthesia procedures conformed to the standards for anaesthesia at our institution. Anaesthesia was induced via the administration of pentothal sodium (5 mg/kg), rocuronium (0.6 mg/kg), and remifentanil (effective site concentration, 2.0–5.0 ng/ml). Anaesthesia was maintained with desflurane (1.0 minimum alveolar concentration), continuous infusion of remifentanil (effective site concentration, 1.0–3.0 ng/ml), and rocuronium (0.3 mg/kg/h). At the end of the operation, muscle relaxation was reversed via the administration of sugammadex (2.0 mg/kg), with recovery defined as a TOF peripheral nerve stimulation ratio > 0.9. Patient vital signs monitored during surgery included heart rate, peripheral oxygen saturation, electrocardiograph, continuous arterial blood pressure, bispectral index, TOF ratio, and end-tidal carbon dioxide concentration. Mechanical ventilation was maintained with a volume-controlled mode of 50% of inspired oxygen fraction. Ventilator settings included a target tidal volume of 8 ml/kg of ideal body weight without PEEP and a respiratory rate of 10–14 cycles/min based on the end-tidal carbon dioxide concentration.

### Measurement of diaphragmatic excursion

Diaphragmatic excursion was measured twice at each time point by a single well-trained expert (K.K.) using a 5–2 MHz convex transducer and an Edge II ultrasound machine (SonoSite, Inc., Bothell, WA), as described previously^3^. The magnitude of the diaphragmatic excursion at each time point was defined as the average of the two measurements. Time points for the measurement of diaphragmatic excursion included before intubation with mechanical mask ventilation in a supine position (T0, bispectral index < 60, TOF ratio > 0.9) with a tidal volume of 8 ml/kg of ideal body weight and a respiratory rate of 12 cycles/min; after intubation with mechanical ventilation (T1, bispectral index < 60, TOF ratio = 0, supine position); 90 min after incision (T2, Trendelenburg position); and at the end of the operation with recovery of muscle relaxation in a supine position (T3, bispectral index < 60, TOF ratio > 0.9) under mechanical ventilation. Diaphragmatic impairment was defined an excursion of diaphragm was < 10 mm under mechanical ventilation^25–27^.

### Statistical analysis and sample size calculation

A pilot study showed that the mean between-group difference in diaphragmatic excursion at T3 was 2.0 ± 2.2 mm. At an α of 0.05 and a power of 0.8, and assuming a 10% dropout rate, 20 patients per group were required.

Baseline characteristics and perioperative variables were compared between the two groups. Continuous variables are expressed as mean ± standard deviation and were compared using Student’s t-tests, whereas categorical variables are expressed as counts and percentages and were compared using the χ^2^ and Fisher’s exact tests. Serial changes in diaphragmatic excursion and pulmonary variables in the two groups were compared by repeated measures of the two-way ANOVA followed by a Bonferroni correction. Data were managed and statistical analyses were performed using IBM SPSS Statistics 21.0 software (IBM, Armonk, NY).

## Data Availability

All data generated or analysed during this study are available from the corresponding author upon reasonable request.
